# Factors associated with admission to intensive care units in COVID-19 patients in Lyon-France

**DOI:** 10.1371/journal.pone.0243709

**Published:** 2021-01-27

**Authors:** Philippe Vanhems, Marie-Paule Gustin, Christelle Elias, Laetitia Henaff, Cédric Dananché, Béatrice Grisi, Elodie Marion, Nagham Khanafer, Delphine Hilliquin, Sophie Gardes, Solweig Gerbier-Colomban, Selilah Amour, Elisabetta Kuczewski, Vanessa Escuret, Bruno Lina, Mitra Saadatian-Elahi, On behalf of COVID-Outcomes-HCL Consortium

**Affiliations:** 1 Service Hygiène, Epidémiologie, Infectiovigilance et Prévention, Centre Hospitalier Edouard Herriot, Hospices Civils de Lyon, Lyon, France; 2 CIRI, Centre International de Recherche en Infectiologie (Equipe Laboratoire des Pathogènes Emergents), Univ Lyon, Inserm, U1111, Université Claude Bernard Lyon 1, CNRS, UMR5308, Lyon, France; 3 Laboratoire de Virologie, Institut des Agents Infectieux, Hôpital de la Croix-Rousse, Hospices Civils de Lyon, Lyon, France; 4 Virpath—Grippe, de l’émergence au contrôle, Centre International de Recherche en Infectiologie (CIRI), Inserm U111, CNRS 5308, ENS, Faculté de Médecine RTH Laënnec, Lyon, France; University of Maryland School of Medicine, UNITED STATES

## Abstract

**Introduction:**

A new respiratory virus, SARS-CoV-2, has emerged and spread worldwide since late 2019. This study aims at analysing clinical presentation on admission and the determinants associated with admission in intensive care units (ICUs) in hospitalized COVID-19 patients.

**Patients and methods:**

In this prospective hospital-based study, socio-demographic, clinical and biological characteristics, on admission, of adult COVID-19 hospitalized patients presenting from the community for their first admission were prospectively collected and analysed. Characteristics of patients hospitalized in medical ward to those admitted in ICU were compared using Mann-Whitney and Chi-square or Fisher exact test when appropriate. Univariate logistic regression was first used to identify variables on admission that were associated with the outcome i.e. admission to an ICU versus total hospital stay in a medical ward. Forward selection was then applied beginning with sex, age and temperature in the multivariable logistic regression model.

**Results:**

Of the 412 patients included, 325 were discharged and 87 died in hospital. Multivariable regression showed increasing odds of ICU hospitalization with temperature (OR, 1.56 [95% CI, 1.06–2.28] per degree Celsius increase), oxygen saturation <90% (OR, 12.45 [95% CI, 5.27–29.4]), abnormal lung auscultation on admission (OR, 3.58 [95% CI, 1.58–8.11]), elevated level of CRP (OR, 2.7 [95% CI, 1.29–5.66for CRP>100mg/L vs CRP<10mg/L). and monocytopenia (OR, 3.28 [95% CI, 1.4–7.68]) were also associated with increasing odds of ICU hospitalization. Older patients were less likely to be hospitalized in ICU (OR, 0.17 [95%CI, 0.05–0.51].

**Conclusions:**

Age and delay between onset of symptoms and hospital admission were associated with the risk of hospitalisation in ICU. Age being a fixed variable, interventions that shorten this delay would improve the prognosis of Covid-19 patients.

## Introduction

Severe acute respiratory syndrome coronavirus 2 (SARS-CoV-2), first detected in December 2019 in the Hubei province of China [1–3], was declared as a pandemic by the World Health Organization on March 11, 2020. Coronavirus disease 2019 (COVID-19) is the emerging infectious disease due to SARS-CoV-2, associated with lower or upper respiratory infection even though less typical clinical features or asymptomatic cases have also been reported [4, 5]. The infection fatality rates of COVID-19 varies considerably, with a median of 0.27% across 32 locations included in a recent analysis of seroprevalence studies in the general population [6]. Crude case fatality rate ranges from 2% to 4% but can reach 12% to 15% in the elderly [7].

The first published cases of SARS-CoV2 infection in Europe were travellers from Wuhan who were tested positive in France (two in Paris and one in Bordeaux) on January 24, 2020 [8]. As of September 24, 2020, 497,230 confirmed cases have been reported in France, including 31, 511 (20, 940 in hospitals) deaths [9]. The Auvergne-Rhône-Alpes region located in the southeast of France has a population of more than 6 million inhabitants. By April 24^th^, 1,287 patients, including 200 patients in ICUs were hospitalized in public and private structures in Rhône and Nord-Isère.

COVID-19 related complications, patient outcomes and mortality rates reported so far have varied considerably between countries most probably owing to differences in healthcare systems and the availability of ICU beds. Moreover, the prevalence of underlying chronic diseases such as obesity and diabetes, known to be important determinants in the clinical course and outcome of COVID-19 [10] are also different throughout the world. In addition, a large number of published reports so far have described hospitalized COVID-19 patients with incomplete data vis-à-vis hospital follow-up because a substantial proportion of patients remained hospitalized at the time of manuscript submission or publication.

Knowledge of the baseline characteristics and outcomes of hospitalized COVID-19 patients from different parts of the world is crucial for the decision-making process at national and international levels in order to properly respond to the pandemic.

The aim of this study was to report the clinical features and outcomes of patients filling the WHO case definition for confirmed COVID-19 and admitted to Lyon university-affiliated hospitals with complete documentation of the hospital stay from February 8 to April 24, 2020. Demographic, clinical and biological characteristics on admission associated with the risk of ICU admission was assessed.

## Methods

### Study design and participants

This prospective, observational, hospital-based study (NOSO-COR, ClinicalTrials: NCT04290780) is an ongoing international multicentre study carried out in France and hospitals affiliated with the GABRIEL network [11]. The latter is a network of hospitals involved in prospective studies on respiratory infection in emerging countries, leaded by Merieux Foundation (www.https://www.gabriel-network.org/). However, the present paper was limited to community-acquired COVID-19 confirmed patients admitted to four university-affiliated hospitals in Lyon (Hospices Civils de Lyon, 5,300 beds).

Any adult patient who presented from the community with an infectious syndrome based on the WHO definition of COVID-19 [12], admitted for the first time to one of the four participating university-affiliated hospitals in Lyon between February 8 and April 24, 2020, and hospitalized for a period of at least 24 hours, was included.

The study was approved by the clinical research and ethics committee of Ile de France V on March 8, 2020 (No. 20.02.27.69817 Cat 3).

### Data collection

Identification of community-acquired confirmed SARS-CoV-2 patients was based on a daily extraction of real-time reverse transcriptase-polymerase chain reaction (RT-PCR) positive patients from the virology laboratory. Electronic medical records were the main source of data collection. Demographic characteristics, underlying comorbidities, clinical, and biological parameters and patient outcome data were collected prospectively on an electronic case-report form designed especially for the purpose of the project. Clinical outcomes were monitored up to hospital discharge or death. All data were double-checked after computerization.

Nasopharyngeal swab samples were collected as part of the standard care in patients presenting signs and symptoms of SARS-CoV-2 infection. Samples were transferred to the French national reference centre of respiratory viruses for the detection of SARS-CoV2 by RT-PCR [13]. Patients with positive RT-PCR results were defined as laboratory-confirmed SARS-CoV-2.

### Statistical analysis

Given the descriptive nature of this observational study and the emergency context, no statistical sample size calculation was performed. Sample size was equal to the number of patients included during the study period.

Continuous variables were reported as median and interquartile range (IQR). Categorical variables were described as frequencies (%). We compared characteristics of patients hospitalized in medical ward to those admitted in ICU using Mann-Whitney and Chi-square or Fisher exact test when appropriate.

Univariate logistic regression was first used to identify variables on admission that were associated with the outcome i.e. admission to an ICU versus total hospital stay in a medical ward. Forward selection was then applied beginning with three variables in the model: sex, temperature and age or delay between onset of symptoms and hospital admission. Variables that were significant at 0.15 levels in univariate analysis were first introduced one by one in turn in the multivariate regression model. Interaction with covariates were tested and the most significant variable was added in the model. This treatment was repeated with the remaining variables until reduction in the deviance between the current and the previous model was still significant at 0.05 level with no excessively large ORs' confidence interval. Goodness of fit of the models was assessed using Hosmer-Lemeshow test (function hoslem.test, R package ResourceSelection). This stepwise multivariable analysis was applied to 321 patients for whom complete biological data (white blood cells, neutrophil, lymphocyte, monocyte, creatinine, red blood cells, haemoglobin, C-reactive protein and oxygen saturation) were available. Statistical tests were 2-tailed with a level of statistical significance of < .05. Statistical analysis was performed using R language version 3.5.2 (https://cran.r-project.org/).

## Results

### Patient characteristics on admission

From February 8 to April 24, 2020, a total of 412 SARS-CoV-2 confirmed patients with known date of hospital discharge or death were included. Overall, 66 patients (16.0%) were admitted directly to ICUs and 320 (77.7%) were hospitalized in medical wards, of whom 26/320 (8.1%) required subsequent transfer to ICUs. Median age was 72.0 years [IQR, 57–83] and 56.3% were men in the overall population. A total of 188 (45.6%) patients were younger than 70 years, 139 (33.7%) were aged between 70 and 84 years old and 82 (20.6%) were older than 85 years. One or more pre-existing comorbidities were present in 286 patients (69.4%): cardiovascular diseases (47.6%), diabetes (19.9%) and chronic lung diseases (15.0%) being the most common. The most frequently reported signs and symptoms on admission were cough (73.5%), dyspnoea/tachypnoea (64.3%), general weakness (61.4%) and fever (>37.8°C, 57.0%). Abnormal lung auscultation was observed in 229 patients (55.6%). Demographic data, clinical signs and symptoms on admission according to hospitalization ward are summarized in Table 1. Males were significantly more prone to hospitalization in ICUs (*P* = .0003). The proportion of patients with comorbidities was similarly distributed among medical wards and ICUs except for cardiovascular diseases (*P* = .04). Diffuse or abdominal pain were reported significantly more often in patients hospitalized in medical wards (*P* = .003 and *P* = .02 respectively). Patients hospitalized in ICUs presented more often with fever (>37.8°C, 66.3% and vs 54.4%; *P* = .04), shortness of breath (77.2% vs 44.1%; *P* < .001), showed more frequently abnormal lung auscultation (71.7% and 76.9% vs 50.9%; *P* = .0005) and suffered from dyspnoea/tachypnoea (85.9% vs 58.1%; *P* < .001). Patients hospitalized in ICU were significantly more likely to have oxygen saturation less than 90%, to receive specific treatment, and to be under ventilation. Duration of symptoms were also significantly higher in ICU hospitalized patients than those in medical ward (P < .001). The time between symptom onset and hospital admission was significantly lower in patients hospitalized in medical wards (median, 6 [IQR, 2–9] vs 7 [IQR,4–10]; *P* = .004).

**Table 1:** Demographic and clinical characteristics on admission of 412 confirmed COVID-19 hospitalized patients at Lyon University Hospitals, France.

	Medical wards (n = 320)	ICU (n = 92)	p-value
**Age(years)**	73 (57–84)*	68.5 (55.8–78)	0.03
Age (≥75 years)	151 (47.2)	33 (35.9)	0.06
<50	54 (16.9)**	15 (16.3)	
50–69	87 (27.2)	32 (34.8)	
70–84	102 (31.9)	37 (40.2)	
> = 85	77 (24.1)	8 (8.7)	
**Gender**			
Male	165 (51.6)	67 (72.8)	0.0003
**Comorbidities**			
^a^Cardiovascular disease	161(50.3)	35 (38)	0.04
Systolic arterial pressure (mmHg)	136 (120–153.2) [292]	132 (115–147) [85]	0.15
Diastolic arterial pressure (mmHg)	78 (67–86) [292]	77 (62–84) [85]	0.15
PAS> = 140 and/or PAD> = 90	142/292 (48.6)	36/85 (42.4)	0.33
Diabetes	64 (20)	18 (19.6)	1
Chronic lung disease	48 (15)	14 (15.2)	1
Renal diseases	39 (12.2)	17 (18.5)	0.12
Malignancy	42 (13.1)	13 (14.1)	0.86
Chronic neurological diseases	46 (14.4)	7 (7.6)	0.11
Liver diseases	21 (6.6)	6 (6.5)	1
Immunodeficiency	20 (6.2)	6 (6.5)	1
**Signs and symptoms at admission**			
Temperature (°C)	38 (37–38.4)	38.3 (37.5–39)	0.0005
Fever (>37.8°C)	174 (54.4)	61 (66.3)	0.04
Fever (>39.0°C)	22 (6.9)	15 (16.3)	0.01
Historic of fever	273 (85.3)	85 (92.4)	0.08
Cough	237 (74.1)	66 (71.7)	0.69
General weakness	199 (62.2)	54 (58.7)	0.55
Shortness of breath	141 (44.1)	71 (77.2)	<0.001
Diffuse pain	107 (33.4)	16 (17.4)	0.003
Diarrhoea	89 (27.8)	24 (26.1)	0.79
Myalgias	71 (22.2)	14 (15.2)	0.19
Headache	55 (17.2)	12 (13)	0.42
Nausea	43 (13.4)	11 (12)	0.86
Runny nose	40 (12.5)	9 (9.8)	0.58
Confusion	27 (8.4)	8 (8.7)	1
Abdominal pain	31 (9.7)	2 (2.2)	0.02
Anosmia	26/305 (8.5)	4/92 (4.3)	0.26
Ageusia	25/305 (8.2)	4/92 (4.3)	0.26
Sore throat	20 (6.2)	4 (4.3)	0.62
Chest pain	12 (3.8)	2 (2.2)	0.74
Joints pain	7 (2.2)	1 (1.1)	0.69
Dyspnoea/tachypnoea	186 (58.1)	79 (85.9)	<0.001
Abnormal lung auscultation	163 (50.9)	66 (71.7)	0.0005
Pharyngeal exudate	21 (6.6)	6 (6.5)	1
Oxygen saturation (%)	95 (92–97) [279]	88 (82.5–93) [71]	<0.001
Oxygen saturation <90%	26/279 (9.3)	38/71 (53.5)	<0.001
Treatment for Covid-19	119/304 (39.1)	70/89 (78.7)	<0.001
Ventilation	18 (5.6)	67 (72.8)	<0.001
**Duration of symptoms**	15 (12–18.8) [198]	22.5 (20.2–27) [34]	<0.001
LOS (Alive)	8 (4–12) [276]	12.5 (9–17.8) [50]	<0.001
LOS (Deceased	8.5 (6–13) [44]	11 (6–18.2) [42]	0.11
**Delays (days) between**			
**Onset of symptoms and hospital admission**	6 (2–9)	7 (4–10)	0.004
< 3 days	84 (26.2)	10 (10.9)	
3–10 days	43 (13.4)	22 (23.9)	
> 10 days	193 (60.3)	60 (65.2)	
**Deceased**	45 (14.1)	42 (45.7)	<0.001

* Median (IQR) for continious variables

** % in paranthese for categorial variables

COVID-19: coronavirus disease 2019; ICU: Intensive care unit; IQR: interquartile range; LOS: length of stay.

*P* < .05 was considered statistically significant

^**a**^Cardiovascular disease included hypertension and heart failure

[n] indicates the patients without missing values for continuous variables

As of April 24, 2020, 87 (21.1%) patients had died. The crude case fatality rate differed between patients hospitalized in medical wards (14.1%), and those hospitalized in ICU (45.7%, P < .0001).

Initial biological data on admission are represented in Table 2. The majority of biological parameters were in the normal range although their values differed between patients admitted to ICUs and those hospitalized in medical wards. Lymphocytopenia and monocytopenia were found in 70.7% and 37.0% of ICU hospitalized patients as compared to 51.9% and 14.6% in medical ward patients respectively (P < .001). Elevated levels of aspartate aminotransferase (AST> 37 U/L), alanine aminotransferase (ALT> 61 U/L), lactate dehydrogenase (LDH> 241 U/L), C-reactive protein (CRP> 100 mg/L) and urea (>6.6 mmol/L) on admission were also noted in ICU patients.

**Table 2:** Laboratory measures on admission of confirmed Covid-19 hospitalized patients at Lyon University Hospitals, France.

		Median (IQR)		
	Reference range	Medical wards (n = 320)	ICU (n = 92)	p-value
**Complete blood count**				
**White blood cells (G/L)**	[4–10]	5.9 (4.5–7.9)* [308]	7.1 (5.2–9.3) [92]	0.002
<4		49/320 (15.9)**	13/92 (14.1)	0.75
>10		259/320 (84.1)	79/92 (85.9)	0.75
**Neutrophils (G/L)**	[1.8–7.5]	4.1(2.8–5.9) [308]	5.5 (3.8–8.2) [92]	<0.001
>7.5		36/320 (11.7)	27/92 (29.3)	0.0001
**Lymphocytes (G/L)**	[1–4]	1 (0.7–1.4) [308]	0.7 (0.6–1.1) [92]	0.0001
<1		160/320 (51.9)	65/92 (70.7)	0.002
**Monocytes (G/L)**	[0.2–0.9]	0.5 (0.4–0.7) [308]	0.4 (0.2–0.5) [92]	<0.001
<0.3		45/320 (14.6)	34/92 (37)	<0.001
**Platelets (G/L)**	[150–400]	195(155–257) [305]	196(152–268.8) [90]	0.98
<150		70/320(23)	21/92(23.3)	1
**Red blood cells (globules rouges)**	[4.0–6.0]	4.6 (4.1–5) [308]	4.6 (4.1–5.1) [92]	0.47
**Haemoglobin (g/L)**	[120–170]	134 (120–146) [308]	137.5 (118.8–147.2) [92]	0.57
<120		74/320 (24)	24/92 (26.1)	0.68
**NLR**		4.2(2.4–7.4) [308]	6.9(4.2–11.7) [92]	<0.001
> = 3.3		186/320(60.4)	76/92(82.6)	<0.001
**PLR**		197.1(135.2–289.3) [305]	253.6(184.7–392.5) [90]	<0.001
> = 180		173/320(56.7)	68/92(75.6)	0.001
**Prothrombin time (%)**	[70–150]	82(68–90) [239]	76(66.2–87) [74]	0.12
≥ 70		175/320(73.2)	47/92(63.5)	0.11
**Inflammation**				
**CRP (C Reactive Protein) (mg/L)**	<5	49.9(18.3–107) [305]	127.2(67.6–178.8) [73]	<0.001
>100		85/320(27.9)	47/92(64.4)	<0.001
>20.3		220/320(72.1)	69/92(94.5)	<0.001
**Biochemical**				
**Creatinine (μmol/L)**	[45–104]	81(63–101) [305]	83(71.5–114) [91]	0.06
>104		69/320(22.6)	26/92(28.6)	0.26
**Urea (mmol/L)**	[2.5–9.2]	6.3(4.5–9) [304]	7(4.8–9.7) [91]	0.1
>6.4		152/320(50)	57/92(62.6)	0.04
**AST (Transaminase ASAT) (U/L)**	[15–37]	39(28–59) [244]	62(45–80) [79]	<0.001
>37		132/320(54.1)	71/92(89.9)	<0.001
**ALT (Transaminase ALAT) (U/L)**	[13–61]	26(17–45.5) [255]	35(23–62.5) [79]	<0.001
>61		33/320(12.9)	21/92(26.6)	0.008
**LDH (U/L)**	[87–241]	313(231.5–402) [111]	408(358.5–496) [23]	0.001
>241		78/320(70.3)	21/92(91.3)	0.04
**Sodium (mmol/L)**	[136–145]	137(134–139) [305]	136(134–138) [91]	0.41
**Potassium (mmol/L)**	[3.5–5.1]	4.1(3.8–4.4) [301]	4(3.6–4.4) [90]	0.3

* Median (IQR) for continious variables

** % in paranthese for categorial variables

COVID-19: coronavirus disease 2019; ICU: intensive care unit; IQR: interquartile range; LOS: length of stay; AST: aspartate aminotransferase, ALT: alanine aminotransferase; LDH: lactate dehydrogenase; CRP: C-reactive protein; NLR: Neutrophils to Lymphocytes Ratio, PLR: Platelets to Lymphocytes Ratio

*P* < 0.05 was considered statistically significant

[n] indicates the patients without missing values for continuous variable

### Patient determinants associated with ICU hospitalization

The analysis was based on 321 patients with complete biological data. The crude case fatality rate was not statistically different between included patients and those not included (20.0% (75/321) vs 31.6%, (12/38); *P* = .26).

The time between the onset of symptoms and hospital admission was inversely associated with age in our study with a shorter time for older patients (*p* < 0.05) (Supplementary material). Due to this strong correlation, an independent effect of each of these variables could not be detected. Demographic, clinical and biological characteristics on admission associated with admission in ICUs are therefore summarized in Table 3 for each of these variables.

**Table 3:** Determinants associated with admission to intensive care units in 321 Covid-19 patients at Lyon University hospitals, France.

**A: With age in the model**			
	Adjusted OR	95%CI	P-value
**Sex**			
Female (17/147)	1		
Male (39/174)	1.66	0.76–3.62	0.2
**Age (years)**			
< 70 (27/144)	1		
70–85 (21/100)	0.75	0.33–1.7	0.49
85–103 (8/77)	0.17	0.05–0.51	0.002
^**a**^**Temperature** Continuous variable	1.56	1.06–2.28	0.02
**Oxygen saturation (%)**			
90–100 (26/265)	1		
<90 (30/56)	12.45	5.27–29.4	<0.0001
**Abnormal lung auscultation**			
No (13/141)	1		
Yes (43/180)	3.58	1.58–8.11	0.002
**C Reactive Protein, mg/L** (Normal range <5 mg/L)			
≤100 (21/217)	1		
>100 (35/104)	2.7	1.29–5.66	0.008
**Monocyte count, x10**^**9**^**/L** (Normal range 0.2–0.9 x10^9^/L)			
≥ 0.3 (36/263)	1		
< 0.3 (20/58)	3.28	1.40–7.68	0.006
**B: With delay between onset and hospital admission in the model**			
	Adjusted OR	95%CI	P-value
**Sex**			
Female (17/147)	1		
Male (39/174)	1.88	0.87–4.10	0.11
**Delay between onset and hospital admission (day)**			
0–2 (5/73)	1		
3–10 (38/200)	5.96	1.65–21.5	0.006
>10 (13/48)	6.90	1.68–28.4	0.007
^**a**^**Temperature** Continuous variable	1.62	1.10–2.39	0.01
**Oxygen saturation (%)**			
90–100 (26/265)	1		
<90 (30/56)	11.48	4.86–27.1	<0.0001
**Abnormal lung auscultation**			
No (13/141)	1		
Yes (43/180)	3.50	1.56–7.85	0.002
**C Reactive Protein, mg/L** (Normal range <5 mg/L)			
≤100 (21/217)	1		
>100 (35/104)	2.01	0.95–4.23	0.07
**Monocyte count, x10**^**9**^**/L** (Normal range 0.2–0.9 x10^9^/L)			
≥ 0.3 (36/263)	1		
< 0.3 (20/58)	2.86	1.26–6.50	0.02

OR: odds ratio, CI: confdence interval

In multivariable logistic regression: i) ICU admission was the outcome and ii) age or delay between onset and hospital admission sex, continuous temperature, oxygen saturation, abnormal lung auscultation, monocyte count and C-reactive protein were additive covariates, Akaike information criterion = 216.0, test of Hosmer and Lemeshow goodness of fit with 10 bins: P = 0.59

^a^odds ratio of ICU admission was multiplied by 1.62 per degree celsius increase

The results of multivariate regression analysis including age (Table 3) showed that older patients (≥ 85-year-old) were less admitted in ICUs (OR, 0.17 [95%CI, 0.05–0.51] as compared to those < 70 years old. Temperature (OR, 1.56 [95%CI, 1.06–2.28] per degree Celsius increase; *P* = .02) and abnormal lung auscultation on admission (OR, 3.58 [95%CI, 1.58–8.11]; *P* = .002) were associated with a higher risk of admission in ICUs. Patients with oxygen saturation < 90% had higher risk of ICU admission (OR, 12.5 [95%CI, 5.27–29.4] compared to those with oxygen saturation ≥ 90%, *P* < 0.001). The odds of ICU admission revealed a statistically significant increasing trend with an elevated level of CRP (OR, 2.7 [95%CI, 1.29–5.66] for CRP> 100mg/L vs CRP < 10mg/L; *P* = .008). Monocytopenia (monocytes < 0.3 G/L) was associated with increased risk of ICU hospitalization (OR, 3.28 [95%CI, 1.4–7.68; P = 0.006]]. When age was replaced by time from onset to hospital admission in the multivariate regression model (Table 3), CRP was not anymore significantly associated with the risk of ICU admission (OR, 2.01 [95%CI, 0.95–4.23] for CRP> 100mg/L vs CRP < 10mg/L; *P* = .07).

## Discussion and conclusions

This report of French hospitalized COVID-19 patients with full follow-up data completes epidemiological information already available from other European countries [14–16]. Overall, 16.6% of the patients were directly admitted to ICUs and 6.3% were transferred to ICUs from medical wards. The study comprised 412 patients with 87 deaths and 325 patients discharged alive.

The observed overall case fatality rate of 21.1% in this series is higher than those reported in China [17, 18], but is similar to what has been observed in New York City [19]. The relatively younger age of patients in the Chinese studies (median ages: 56 and 49 respectively) could lead to less sever disease that explain the lower mortality rates reported in these studies.

Mortality rates of almost 46% in our patients hospitalized in ICU was higher than that of 26% reported in ICU-hospitalized patients in Lombardy [14]. However, at the time of reporting, 58% of patients included in the latter study were still hospitalized. In addition, the attributable mortality related to Covid-19 could not be assessed in our patients who had several underlying comorbidities that could contribute to the observed mortality rate.

The most commonly known manifestations of the disease i.e. cough, weakness and fever on admission in our patients were in general similar to those reported in other studies [17, 20, 21]. As reported earlier, cardiovascular diseases and diabetes were the most common comorbidities [18, 22].

In agreement with the results of a recent single-arm meta-analysis [23], men accounted for a higher proportion of COVID-19 patients than women in the present study. Similar findings have been reported for MERS-CoV [24]. Women and men traditionally differ in their perceptions of risk [25]. In women, better adoption of protective behaviours such as hand-washing [26], in particular in the context of a pandemic [27], could at least in part explain the observed results. Differences in underlying comorbidities, in particular lower cardiovascular diseases in women could also explain higher rate of ICU admission in men. However, the rates of patients with cardiovascular diseases were not different between the two genders in our study.

Consistent with respiratory viral infections, our hospitalized patients had lymphopenia and elevated levels of LDH and CRP. These laboratory abnormalities were found more often in patients hospitalized in ICU. Lymphopenia and increased levels of LDH and CRP were also reported in the meta-analysis of 1994 COVID-19 patients [21].

Our results suggest that temperature, abnormal lung auscultation on admission, high levels of CRP, and monocytopenia could increase the risk of ICU hospitalization. A high level of CRP has been reported to be an independent risk factor to assess the severity of COVID-19 [28].

We found that older age was associated with lower risk of ICU admission. By comparison with the known severity of influenza in elderly and due to the lack of sufficient knowledge about the consequences of COVID-19 on elderly during the first wave of the pandemic in France, this population could present to the hospital as soon as the onset of symptoms. Rapid management and start of appropriate treatment thanks to earlier presentation at hospital could explain the lower rates of ICU hospitalization observed in older patients. The risk of reduced survival after ICU-related invasive treatment in this fragile population could also explain, at least in part, the observed inverse relationship between age and ICU admission [20]. Finally, in the context of a pandemic, shortage of intensive care resources could impact the decisions about the most appropriate treatment.

The time between the onset of symptoms and hospital admission was strongly associated with ICU admission and could be influenced by multiple determinants such as socio-economic status, personal risk perception, and access to care. This delay could be considered as a warning marker to alert medical providers on the possibility of critical illness when patients present later in their disease course.

The prospective design, inclusion of both severe and non-severe cases and complete follow-up of the study population are the main strengths of the present study. Multivariable analysis was based on 374 patients with complete biological data. However, selection bias remains low since the case fatality rate did not differ between patients who were included and not included in the model. Only biological measures on admission were analysed because repeated measurements were most likely only performed in more severe cases.

In conclusion, age and delay between onset of symptoms and hospital admission were associated with the risk of hospitalisation in ICU. Age being a fixed variable, interventions which shortened this delay would improve the prognosis of Covid-19 patients.

## Supporting information

S1 File (XLSX)

S2 File Distribution of the time between the onset of symptoms and hospital admission by age category. (DOCX)
