# Supplementary material for: Factors associated with admission to intensive care units in COVID-19 patients in Lyon-France
Source: PLoS One. 2021 Jan 27;16(1):e0243709. doi: 10.1371/journal.pone.0243709 (PMC7840037; doi:10.1371/journal.pone.0243709)
Supplement: S2 File — (DOCX) [file pone.0243709.s002.docx]

Distribution of the time between the onset of symptoms and hospital admission by age category

**Time between onset of symptoms and hospital admission (days)**

**Age (years)**


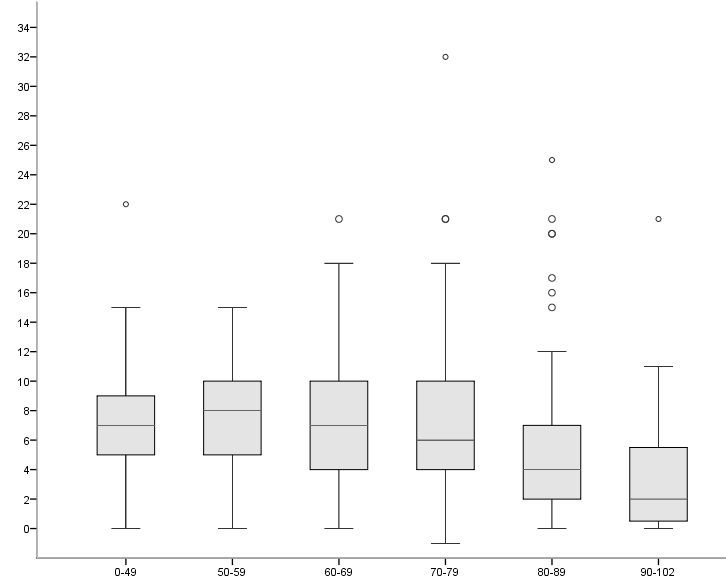


Anova: *P* < 0.0001

Kruskal-Wallis: *P* < 0.0001

Spearman (Age as continuous variable): *P* < 0.0001
